# Complex relational needs impede progress in NHS Talking Therapies (IAPT): implications for public mental health

**DOI:** 10.3389/fpubh.2023.1270926

**Published:** 2023-10-02

**Authors:** Orestis Zavlis

**Affiliations:** ^1^Department of Psychiatry, University of Oxford, Oxford, United Kingdom; ^2^Complex Needs Service, Oxford Health NHS Foundation Trust, Oxford, United Kingdom

**Keywords:** personality disorder, complex relational needs, NHS Talking Therapies, IAPT, depression and anxiety

## Introduction

Although influential in “*Increasing Access to Psychological Therapies”* (IAPT), the IAPT initiative is predicated on at least two assumptions (namely, homogeneity of clinical pathology and level of severity) that have been recently challenged. A growing corpus of data, for instance, suggests that IAPT cohorts are not homogenous—in neither their severity nor presentation of psychopathology (1). Instead, IAPT cohorts appear to be confounded by a constellation of severe, and usually unrecognized, mental health issues [e.g., (2–4)]. In this paper, I argue that one such issue is *complex relational needs*^1^ and conclude by sketching several ways of addressing this problem.

## Increasing access to psychological therapies (IAPT)

Before exploring the impact of complex needs in IAPT cohorts, it is worth briefly outlining the rationale behind IAPT. As its name implies, the United Kingdom's (UK) IAPT initiative (now known as NHS Talking Therapies) was put forward to “*increase access to (low-intensity) evidenced-based talking therapies”* for “*common mental health issues”* (namely, depression and anxiety) in the British population [see Clark (5) for a detailed overview]. Apart from the obvious psychological benefits, a key aspect of this model was that such benefits could, in fact, pay for their implementational costs (for instance, through decreased medical costs, more taxes from return to work, and increased work productivity) [see Layard et al. (6)]. It must be noted that the IAPT model has been influential in this regard: in a healthcare system funded through general taxation, more than a million people access psychological therapies for depression/anxiety per year (NHS Digital, 2020); extensive analyses on publicly available data showcase the clinical effectiveness of these interventions (7); and economic analyses approve their cost-benefit trade-off (8).

At the same time, however, the IAPT model exhibits a crucial limitation: sometimes, its psychological interventions do not match the level of complexity and/or severity of its clientele [see Martin et al. (1)]. Paradoxically, this limitation is supposed to be a feature, not a “bug”, of the IAPT model. Indeed, central to IAPT is the notion that mental health services are best delivered in a *stepped-care* fashion: less severe cases of “common” psychopathology (like depression and anxiety) are managed by IAPT, while more “extreme” cases of severe psychopathology (like psychosis or bipolar disorder) are referred to and managed by specialist services (for instance, secondary care, at best, or acute admission, at worst) (5). Recent reports, however, cast doubt on the effectiveness of this stepped-care approach to mental health service-delivery. For instance, comprehensive clinical assessments have detected many cases of severe mental health conditions in IAPT services, with as many as 35% exhibiting clinically significant psychotic experiences; 61% scoring above the screening threshold for bipolar disorder; and 69% being at-risk for “personality disorder” (2–4).

Commenting on the nature of all such cases, as well as the reasons why they go unnoticed in IAPT services, is beyond the scope of the current report. Instead, this brief report aims to focus on personality psychopathology (aka complex needs) (see text footnote 1); its inherent, yet unappreciated, intersection with common psychopathology; as well as ways via which its confounding effects on IAPT interventions could be subdued.

## Complex needs in NHS talking therapies

The case that complex relational needs impede progress in IAPT cohorts will be made via two arguments: one philosophical and another empirical. The philosophical argument is as follows: because complex personality pathologies intersect strongly with common psychopathologies (see next paragraphs), it is probabilistically inevitable that the former will confound several treatment attempts of the latter—including, of course, IAPT ones.

To support this philosophical claim, one only has to take a close look at the history and epidemiology of personality conditions. Historically, the conception of “personality” disorders as separate from “mental” disorders (first expressed in 1980, with the introduction of the Diagnostic and Statistical Manual of Mental Disorders 3rd edition; DSM-III) has always been a matter of great controversy. Early researchers argued that such a separation represents a *false dichotomy*, for every mental disorder is at least to some extent a function of maladaptive personality traits [e.g., (9, 10)]. An extreme position of this argument entailed that such maladaptive traits are the *sine qua non* for all common psychopathology—with, for instance, generalized anxiety disorder being a “*pure manifestation of trait anxiety”* [(11), p. 422] or major depression being the outcome of a “*neurotic personality”* [see (12), p. 62]. A more moderate and contemporary perspective, however, is espoused by the 11^th^ edition of the International Classification of Diseases (ICD-11), which places personality issues on a spectrum (ranging from “mild” to “moderate” and “severe”) and recognizes their frequent co-expression with common psychopathologies.

Indeed, recent findings support this contention. Epidemiologically, for instance, the most recent meta-analyses suggest that comorbid personality pathologies are evident in about half of the patients with depression (13) and similarly in around 35–50% of those with anxiety disorders (14). Developmentally, anxiety and depression can sometimes precede (and foster) the onset of “personality disorder”; or be common psychiatric sequala thereof (15). Finally, clinically, the frequent confounding effects of personality difficulties on cases of depression and anxiety have always been acknowledged—to the point where modern psychodynamic accounts make a distinction between *anaclitic depression* (which is more *inter*personal and akin to borderline personality disorder in that it features abandonment fears and interpersonal hypersensitivity) and *introjective depression* (which is more *intra*personal and features low self-worth and high self-criticism) (see Luyten et al. (16), Chapter 7).

*Ergo*, based purely on the laws of probability, it seems highly probable that heterogenous cases of amalgamated personality and common psychopathology could present in IAPT services. But is there direct evidence to support this conclusion?

To the best of my knowledge, there exist at least four studies to support this logical claim.^2^ The first study was by Goddard et al. (17), who sought to examine whether personality difficulties affected clinical outcomes in a large (*N* = 1,249) IAPT sample, using SAPAS (that is, “Standardized Assessment of Personality—Abbreviated Scale”), a well-validated tool for quantifying personality difficulties and detecting cases of “personality disorder”(when SAPAS ≥ 3) with 81% classification accuracy (18). Using SAPAS, Goddard et al. (17) were the first to show that personality issues are indeed present in IAPT and confound treatment outcomes by robustly predicting clinical caseness at the end of IAPT interventions.

Aiming to extend these findings, Mars et al. (19) examined the specificity of similar personality effects on a larger IAPT cohort (*N* = 3,689). Moving beyond SAPAS sum-scores, Mars et al. (19) revealed that the most impactful personality difficulties were long-lasting, *relational* ones (namely, “*forming and maintaining relationships,” “being a loner,”* and “*being dependent on others”*) that are not easily addressed within IAPT settings.

Notably, similar themes emerged in a qualitative study, which revealed that patients (N = 22) with high-risk for personality disorder (SAPAS ≥ 3) reported *relational difficulties* (that is, problems in forming and maintaining relationships) to be their most debilitating symptoms and believed that such complex relational needs cannot be addressed through IAPT's highly standardized and impersonalized cognitive-behavioral treatments (20).

Finally, the high prevalence of complex needs in IAPT was directly confirmed by Hepgul et al. (2), who revealed that a staggering 69% of their representative IAPT sample (*N* = 147) had a high-risk for “personality disorder” (SAPAS ≥ 3) and a 16% met the DSM-IV criteria for “borderline personality disorder”.

On balance, therefore, evidence suggests that the presence and confounding effect of complex relational difficulties on IAPT cohorts is not merely a logical possibility, but, more so, a frequent clinical reality. Yet currently, no guidelines exist for the detection or treatment of such difficulties in IAPT services. This service gap is particularly concerning, since comorbid personality difficulties are known to: (1) increase the likelihood of individuals dropping out of therapy (21, 22) and (2) confound treatment outcomes for both depression (23) and anxiety conditions (24, 25). Indeed, UK national data suggest that: (1) of the 1,647,716 IAPT referrals in 2019/20, 63.21% did not complete treatment and (2) of those who completed treatment, around 60% did not achieve clinical recovery (26). Although speculative, it may not be farfetched to assume that many such cases of treatment resistance are due to comorbid personality difficulties (among other comorbidities, of course).^3^

## Discussion

In this paper, I have argued that unrecognized personality (or, more specifically, relational) difficulties impede progress in IAPT cohorts, by highlighting the inherent, yet unappreciated, interface of personality and common psychopathology. In the remainder of this Discussion section, I explore three possible ways forward in light of these issues.

First, I (tentatively) propose SAPAS to be introduced as a brief screening tool for probable personality conditions. As forewarned is forearmed, equipping IAPT clinicians with a brief assessment of relational difficulties could help them identify and accordingly intervene on cases of (mild/moderate/severe) ‘personality disorder'—either by more appropriately formulating those complex needs or by referring them to higher-intensity interventions.

Even if personality difficulties are explicitly recognized and clinically formulated, though, they may still remain unaddressed in response to standard IAPT interventions. Thus, a second way forward might be to introduce psychotherapeutic interventions specifically for personality difficulties in IAPT services. Although national guidelines caution against the use of low-intensity treatments for individuals with “personality disorder,” recent feasibility trials have pointed toward the opposite conclusion (28)—with some low-intensity treatments, such as *structured psychological support*, showing particular promise as cost- and treatment-effective supplements to higher-intensity interventions (29). Assuming these lines of inquiry come to fruition, low-intensity interventions that target more *relational*, as opposed to “*common*”, psychopathology could be incorporated in IAPT services.

Of course, such service expansions should be made with caution so as not to contribute to the increasing “*fragmentation”* and “*insulation”* of healthcare provision (which IAPT has, in fact, compounded) (30). In light of this, a final way forward could include balancing service expansion and integration. As an example, in a UK case study, the integration of disparate primary care services (such as IAPT therapists, general practitioners, and nurses) into a single “*care network”* led to more carefully considered referrals and better recovery rates for patients (31). Arguably, a similar integration of primary and specialist services for personality disorder could lead to better recognition and treatment of clinical populations who suffer its mild-to-moderate form.

Thus far, such populations have been largely neglected, given archetypal paintings of “personality disorder” as a severe psychopathology. Recognizing, however, that personality psychopathology lies on a spectrum and is far more common than once thought (up to 10% population prevalence) could help create treatment pathways for those who suffer its less severe form (27).

## Author contributions

OZ: Writing—original draft, Writing—review and editing.

## Footnotes

^1^Throughout this paper, I use the term “complex (relational) needs” synonymously with the ICD-11 conception of “personality disorder” as a monolith. The former term is preferred over the latter, not least because it is less stigmatizing, but more so because it emphasizes the relational issues of personality conditions (which appear to be the most impactful at least in IAPT cohorts).

^2^To identify these studies, I scrutinized the most recent systematic reviews [e.g. (1)] on this topic for relevant references; checked their cross-references; and performed a literature search on PsycInfo [using {Personality Disorder} OR {Personality Difficulties} OR {Relational Difficulties} AND {IAPT} as search terms in title/abstract, for the time period of 2008 (inception of IAPT) to 2022], yielding nine studies from which four were deemed pertinent.

^3^Although the argument at hand is rather UK centric, it could be extended to other countries that have adopted IAPT (or IAPT-like) services (such as Norway, Sweeden, or Australia) by raising the strong possibility that many such primary care services are plagued by unrecognized personality psychopathologies [see Tyrer and Mulder (27), Chapter 7].
